# Convulsions of Children

**Published:** 1836

**Authors:** 


### III.—Convulsions of Children.

It is well known, that children of various ages, but especially while they are under six or eight years, are liable to convulsions from causes which are far from being very obvious, and that the first attack is often fatal. Worms, constipation, a surfeit or plethora with cerebral determination, is generally assigned as the immediate cause of the attack. An emetic, the lancet, and a tepid bath, are commonly the first remedies. But during the convulsion, the child cannot swallow; blood-letting as we know from experience, (however far it may be carried) will not always secure a favorable termination; and the value of tepid bath, appears to us to be greatly over rated. Discouraged as to a reliance on these means, we lately resolved, on a course somewhat the reverse. Being hastily called to a little girl four years old, (whose sister had expired of convulsions in the bathing tub the year before, immediately after venesection,) we found her in violent spasms. A warm bath had been prepared, but in place of it, we resorted to the cold dash, and in less than a minute, perfect relaxation with quiet of the muscular system, was the consequence.—Lest the sinuses of the brain should have become engorged, we drew a moderate quantity of blood, and then gave repeated enemata. Still further, after the patient was able to swallow we administered an emetic. Nothing, however, was discharged from the alimentary canal, that indicated morbid state of its contents, and it seemed to us, that the victory was won by the cold water alone. It is to bear testimony in favor of this practice, (not suggested as new,) that we have thought the case entitled to publication.

